# Gene jigsaw: Decrypting the CPAMD8 puzzle in Chinese patients with anterior segment dysgenesis

**DOI:** 10.1016/j.gendis.2025.101523

**Published:** 2025-01-16

**Authors:** Yan Liu, Yinuo Wen, Linghao Song, Xinyue Wang, Min Zhang, Zexu Chen, Zhangrui Chen, Nan Yang, Tianhui Chen, Yongxiang Jiang

**Affiliations:** a Eye Institute and Department of Ophthalmology, Eye & ENT Hospital, Fudan University, Shanghai 200031, China; b Key laboratory of Myopia and Related Eye Diseases, NHC; Key Laboratory of Myopia and Related Eye Diseases, Chinese Academy of Medical Sciences, Shanghai 200031, China; c Shanghai Key Laboratory of Visual Impairment and Restoration, Shanghai 200031, China

Anterior segment dysgenesis (ASD) represents a complex spectrum of ocular disorders linked to various genetic mutations, including *PITX3*, *FOXE3*, *BMP4*, *CHRDL1*, *LTBP2*, and *CYP1B1*.^1^ This heterogeneity disrupts the anterior segment's structure, impacting components crucial for vision, including the cornea, iris, and lens, alongside the aqueous humor outflow system. Such disruptions account for the phenotypic diversity observed in ASD, underlining the importance of understanding the genotype-phenotype correlations within this syndrome.^2^^,^^3^

Among the many genes associated with ASD, research on *CPAMD8* (C3 and pregnancy zone protein-like alpha-2-macroglobulin domain-containing 8) mutations has been relatively limited, but its association with severe ocular diseases such as ectopia lentis and glaucoma has garnered increasing attention. The protein encoded by the *CPAMD8* gene is primarily expressed in the non-pigmented epithelium of the ciliary body, suggesting that it plays a crucial role in maintaining the integrity of aqueous humor circulation and zonule structure.

This study included 175 patients who presented to the Eye and ENT Hospital of Fudan University between May 2023 and May 2024 due to ectopia lentis. Through genetic testing, eight Chinese patients with compound heterozygous or homozygous mutations in *CPAMD8* were identified (Table S1). We focused on analyzing the anterior segment structural abnormalities in these patients, aiming to explore the association between *CPAMD8* mutations and ASD, and to provide additional data support for future genotype-phenotype studies.

Comprehensive ophthalmic evaluations were confirmed by two experienced ophthalmologists. Genomic DNA was analyzed using next-generation sequencing of 289 genes associated with inherited anterior eye diseases. The study received ethical approval, and informed consent was obtained from all participants. We used the Kruskal–Wallis test and applied the Benjamini-Hochberg method to adjust the *P*-values, controlling the false discovery rate. Statistical analyses were performed using StataMP 17 and IBM SPSS statistics 20, establishing a significance threshold at *P* < 0.05.

The number of ASD-causing gene mutations in the Human Gene Mutation Database (HGMD) is shown in Figure 1Aa. In addition to the lens, our cohort also exhibited developmental abnormalities in key ocular structures, including the iris, cornea, and ciliary body, without any systemic complications. All affected patients presented with iris dysgenesis, including iris atrophy (11/15 eyes) and posterior iris bowing (9/15 eyes), alongside other ocular anomalies such as megalcornea (13/15 eyes) (Fig. 1B). Four patients developed early-onset cataracts, and four were diagnosed with glaucoma at a relatively young age. Furthermore, two patients exhibited lens thickening due to 360° zonular laxity (Table S2).

**Figure 1:**
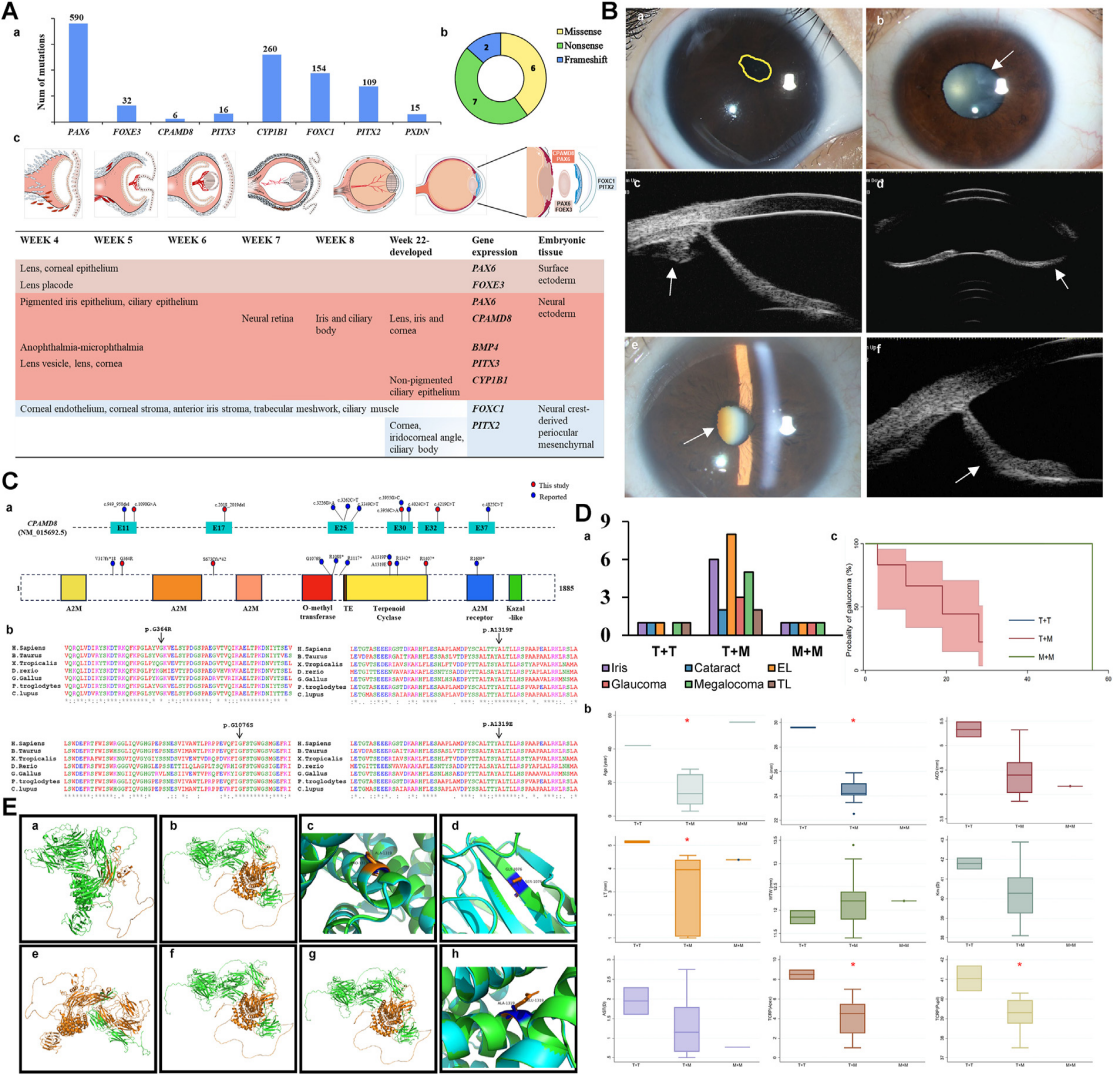
Genotypes and phenotypes of *CPAMD8* mutation. **(A)** CPAMD8's spatiotemporal distribution. (a) Anterior segment dysgenesis (ASD)-causing gene mutations in the Human Gene Mutation Database (HGMD). (b) Percentage distribution of mutation types of CPAMD8 in this cohort. (c) Origin of tissue of the anterior segment and sites of expression of important genes (week 4 to completion of development), mainly affecting the structures of the anterior segment of the eye, including *PAX6*, *PITX2*, *FOEX3*, and *CPAMD8*. Colors represent different embryonic germ layers, and gradients indicate the level of gene expression. **(B)** Representative clinical images of probands with *CPAMD8* mutations. (a) Slit-lamp examination revealed superior displacement of a constricted and irregularly shaped pupil in the right eye of patient 3 (highlighted in yellow). (b) A mild and cloudy cataract was identified in the peripheral cortex in patient 1. (c) Severe nuclear opacity of cataract and irregular-shaped pupil in the right eye of patient 5. (d) Thin ciliary body and ciliary body cysts were observed under ultrasound biomicroscopy in the right eye of patient 3. (e) The anterior chamber is medium-deep, subluxated lens was spherical, and the iris is thin and concave in the left eye of patient 3. (f) The left eye of patient 5 exhibits heightened echogenicity in both eyes, posterior iris bowing, closure of the temporal chamber angle, and an absence of zonular fibers of the lens in all directions. **(C)** Schematic of *CPAMD8* gene and CPAMD8 protein. (a) Schematic of the genomic and protein structure of CPAMD8. Positions of *CPAMD8* mutations identified in this study are marked in red, and positions of previously reported *CPAMD8* mutations by our team are marked in blue. (b) Protein sequence alignments of CPAMD8 among various vertebrate species. The affected amino acids are boxed out. Four missense mutations were highly conserved across species. E, exon; A2M, alpha-2-macroglobulin domain; TE, thioester site. **(D)** Phenotype characterization and genotype-phenotype analysis of *CPAMD8* mutations. (a) The percentage of ocular comorbidities in the T + T, T + M, M + M, and Total groups. (b) Relationship between *CPAMD8* mutations and ocular parameters. (c) Kaplan–Meier estimated probabilities of *CPAMD8* mutations on glaucoma risk according to the age of diagnosis. Iris abnormalities included iris anterior synechia, corectopia, iridonesis, iris hypoplasia, and posterior iris bowing. a: *P* < 0.05, Fisher's exact test. b: *P* < 0.05, Kruskal–Wallis test. AL, axial length; AST, corneal astigmatism; EL, ectopia lentis; Km, mean corneal keratometry; T + T, truncation and truncation variants; T + M, truncation and missense variants; M + M, missense and missense variant. **(E)** Predicted effect of *CPAMD8* mutations on structure–function properties. (a) The orange region is the deletion of amino acids at positions 1609–1885 (p.R1609∗). (b) The orange region is the deletion of amino acids at positions 1117–1885 (p.R1117∗). (c) Superposition before and after mutation, with orange representing the mutant type and green representing the wild type (p.A1319P). (d) Superposition before and after mutation, with orange representing the mutant type and green representing the wild type (p.G1076S). (e) The orange region is the deletion of amino acids at positions 270–1885 (p.V270∗). (f) The orange region is the deletion of amino acids at positions 1342–1885 (p.R1342∗). (g) The orange region is the deletion of amino acids at positions 1088–1885 (p.R1088∗). (h) Superposition before and after mutation, with orange representing the mutant type and green representing the wild type (p.A1319E).

Comprehensive ophthalmic examinations of 15 eyes revealed several characteristic findings, including increased corneal astigmatism (1.40 ± 0.74 diopters) and deeper anterior chambers (4.47 ± 0.52 mm). In addition, the lens thickness in this cohort was 4.13 ± 0.52 mm, the corneal curvature was 40.10 ± 1.37 mm, and the white-to-white measurements were 12.14 ± 0.36 mm (Table S2).

In total, the genetic analysis identified 11 *CPAMD8* mutations (5 nonsense mutations, 4 missense mutations, and 2 frameshift mutations), among which five were novel (Fig. 1Ca).

To increase the testing power, integrating data from other studies, a total of 23 patients (45 eyes) were included in the statistical analysis (Fig. 1Da). Iris abnormalities were the most common ocular complication, affecting 34.40% (43 eyes), followed by ciliary body protrusion, which accounted for 18.40% (23 eyes). We classified patients into three groups based on the types of biallelic mutations they carried, namely M + M (missense + missense), T + M (truncation + missense), and T + T (truncation + truncation), to compare the relationship between mutation types and clinical phenotypes. Cataracts were most common in the M + M group (28.30%, *P* = 0.01), while ectopia lentis was most frequent in the T + M group (25.00%). Glaucoma and iris abnormalities, as fundamental manifestations of ASD, were common among most patients, highlighting the necessity for monitoring ectopia lentis in individuals with *CPAMD8* truncating mutations (Fig. 1Da). Patients in the T + T group demonstrated a later age of onset (*P* = 0.002) and longer axial length (*P* = 0.022). Patients in the T + T group exhibited higher mean keratometry (*P* = 0.011) and total corneal refractive power (Apex *P* = 0.010; Pupil *P* = 0.010). Additionally, this group exhibited larger anterior chamber depth and corneal astigmatism compared with those in the T + M group, although no statistical differences (Fig. 1Db). The lack of significant differences in the age of glaucoma onset among different genotypes suggests that genetic factors may influence specific ocular features rather than the timing of secondary complications (Fig. 1Dc).

Protein alignment confirmed that all missense mutation sites were highly conserved across vertebrate species, reinforcing their likely pathogenicity (Fig. 1Cb**)**. Of the identified variants, five were predicted to result in early protein truncation, indicating a loss-of-function mechanism. The remaining four missense mutations were predicted to impair protein function (Fig. 1E). The phenotypic diversity observed among patients is likely attributable to the spectrum of deletions and the specific functional domains affected. Genotype analysis indicated that biallelic truncating mutations were more commonly associated with more severe and early-onset phenotypes. In contrast, biallelic missense mutations appeared to have a less pronounced effect on aqueous humor outflow.

While the precise function of CPAMD8 remains elusive, it is established that mutations in this gene adversely affect peptidase regulation and significantly impact eye development.^4^ The C-terminal region of CPAMD8 contains a Kazal-like domain, which possesses serine protease inhibitors. It has the potential to antagonize transforming growth factor-beta (TGF-β) and disrupt the bone morphogenetic protein (BMP) signaling pathways, which is essential for ocular development. In this study, patient 4 was identified with c.4024C > T (p. R1342∗) mutation, located in a gene region associated with terpenoid cyclase activity. Bonet-Fernández et al have confirmed the function of CPAMD8 as a protease inhibitor and its role in extracellular matrix remodeling.^1^ It is essential for the integrity of the zonule ligament amidst continuous exposure to matrix metalloproteinases and aqueous humor. Additionally, CPAMD8 actively participates in extracellular matrix restructuring and facilitates the transport of aqueous humor.^1^

Mutations in *CPAMD8* have been identified to impact eye development, exhibiting differential detection levels at distinct developmental stages (Fig. 1Ac).^5^ Preceding the 9th week of embryonic development, CPAMD8 shows elevated levels in the retina. After the 9th week, there is a gradual increase in the detection levels within the lens. By the 22nd week, CPAMD8 had a noticeable presence in the cornea and iris. In adults, *CPAMD8* exhibits the highest expression in the ciliary body and corneal epithelium, suggesting a continued role in maintaining ocular health.^4^ When mutated, normal ocular development might be disturbed.

This study identifies new mutation sites and phenotypic features associated with *CPAMD8* mutations, significantly advancing our understanding of ASD and the rare *CPAMD8* gene. Exploring the clinical and molecular characteristics of *CPAMD8* mutations has deepened insights into the genetic landscape of ASD. Early genetic screening could facilitate timely diagnosis, allowing for closer monitoring and early intervention for high-risk individuals. Furthermore, genotype-phenotype studies may enable precision medicine tailored to patients with different genetic profiles. In this cohort, all missense mutations were associated with milder phenotypes compared with truncating mutations. The patients in the T + M group were more likely to exhibit ectopia lentis, while those in the M + M group were more prone to cataracts. However, expanded association studies and functional analyses are urgently needed to comprehensively understand these relationships and improve clinical outcomes.

## Ethics declaration

The institutional review board approved the study as an extension of our randomized controlled trial (ChiCTR2000039132). All patients signed standard consent forms including consent for data privacy.

## Funding

This study was supported by the 10.13039/501100001809National Natural Science Foundation of China (No. 82070943), the 10.13039/501100001809National Natural Science Foundation of China (No. 82271068), and the 10.13039/501100018625Shanghai Science and Technology Commission of China (No. 22Y11910400).

## CRediT authorship contribution statement

**Yan Liu:** Writing – original draft, Visualization, Methodology, Formal analysis, Data curation, Conceptualization. **Yinuo Wen:** Formal analysis, Data curation. **Linghao Song:** Formal analysis, Data curation. **Xinyue Wang:** Formal analysis, Data curation. **Min Zhang:** Methodology, Investigation, Formal analysis. **Zexu Chen:** Methodology, Investigation. **Zhangrui Chen:** Data curation. **Nan Yang:** Data curation. **Tianhui Chen:** Writing – review & editing, Visualization, Supervision, Methodology, Formal analysis, Conceptualization. **Yongxiang Jiang:** Writing – review & editing, Methodology, Funding acquisition, Conceptualization.

## Data availability

Some or all data, models, or codes generated or used during the study are available from the corresponding author by request.

## Conflict of interests

All authors declared no conflict of interests in this study.
