# Supplementary material for: Gene jigsaw: Decrypting the CPAMD8 puzzle in Chinese patients with anterior segment dysgenesis
Source: Genes Dis. 2025 Jan 16;12(5):101523. doi: 10.1016/j.gendis.2025.101523 (PMC12163380; doi:10.1016/j.gendis.2025.101523)
Supplement: Multimedia component 1 [file mmc1.docx]

**Table S1. *CPAMD8* variants associated with anterior segment dysgenesis in our cohort.**

| **Proband ID** | **Nucleotide  Changes** | **Protein  Changes** | **Exon** | **Heredity** | **Effect** | **Mutation  Taster** | **Domain** | **ACMG** | **Reference** |
| --- | --- | --- | --- | --- | --- | --- | --- | --- | --- |
| **Patient 1** | c.3349C>T | p.R1117* | exon25 | het, AR | nonsense | DC | NA | LP | Reported |
| **Patient 1** | c.949_950delAC | p. V317fs*18 | exon10 | het, AR | frameshift | DC | NA | LP | Reported |
| **Patient 2** | c.3955G>C | p. A1319P | exon30 | het, AR | missense | Poly | Terpenoid Cyclase | VUS | Reported |
| **Patient 2** | c.4825C>T | p.R1609* | exon37 | het, AR | nonsense | DC | NA | P | Reported |
| **Patient 3** | c.4825C>T | p.R1609* | exon37 | het, AR | nonsense | DC | NA | P | Reported |
| **Patient 3** | c.3226G>A | p. G1076S | exon25 | het, AR | missense | DC | O-methyl transferase | VUS | Reported |
| **Patient 4** | c.4024C>T | p.R1342* | exon30 | het, AR | nonsense | DC | NA | P | Reported |
| **Patient 4** | c.3956C>A | p. A1319E | exon30 | het, AR | missense | Poly | Terpenoid cyclase | VUS | Novel |
| **Patient 5** | c.3226G>A | p. G1076S | exon25 | homo, AR | missense | DC | O-methyl transferase | VUS | Reported |
| **Patient 6** | c.3262C>T | p.R1088* | exon25 | homo, AR | nonsense | DC | NA | P | Reported |
| **Patient 7** | c.3956C>A | p. A1319E | exon30 | het, AR | missense | Poly | Terpenoid cyclase | VUS | Novel |
| **Patient 7** | c.2018_2019del | p. S673Cfs*42 | exon17 | het, AR | frameshift | DC | NA | LP | Novel |
| **Patient 8** | c.1090G>A | p. G364R | exon11 | het, AR | missense | DC | NA | VUS | Novel |
| **Patient 8** | c.4219C>T | p.R1407* | exon32 | het, AR | nonsense | DC | Terpenoid cyclase | LP | Novel |

Abbreviations: DC, disease causing; Poly, polymorphism; P, pathogenic; LP, Likely pathogenic; VUS, Variant of Uncertain Significance; Reported, reported in our previously study. NA, not available.

**Table S2. Clinical details of the eight probands with *CPAMD8* mutations.**

| **Patient ID** | **Age** | **Sex** | **Eye** | **AL (mm)** | **ACD (mm)** | **LT**  **(mm)** | **WTW (mm)** | **Km (D)** | **AST (D)** | **TCRP**  **(Apex)** | **TCRP**  **(Pupil)** | **BCVA (LogMAR)** | **A-Iris** | **B-Iris** | **Cata** | **EL** | **Glau** | **Meg** | **TL** |
| --- | --- | --- | --- | --- | --- | --- | --- | --- | --- | --- | --- | --- | --- | --- | --- | --- | --- | --- | --- |
| **Patient 1** | 25 | F | R | 24.98 | 4.83 | 3.30 | 12.5 | 40.8 | 0.60 | 40.30 | 40.30 | 0.00 | + | - | + | + | - | + | - |
| **Patient 1** | 25 | F | L | 24.31 | 4.31 | 4.11 | 12.2 | 41.1 | 2.00 | 40.10 | 40.00 | 0.00 | + | - | + | + | - | + | - |
| **Patient 2** | 7 | F | R | 24.06 | 4.09 | 4.19 | 12.2 | 39.1 | 0.60 | 38.80 | 38.90 | 0.05 | + | + | - | + | - | + | - |
| **Patient 2** | 7 | F | L | 24.07 | 4.13 | 4.23 | 11.7 | 39.4 | 0.70 | 38.90 | 38.90 | 0.10 | + | + | - | + | - | + | - |
| **Patient 3** | 3 | F | R | 22.53 | 3.89 | 4.46 | 12.2 | 38.1 | 0.50 | 37.00 | 37.50 | 0.05 | + | + | + | + | + | + | + |
| **Patient 3** | 3 | F | L | 24.28 | 3.94 | 4.42 | 11.8 | 39.0 | 1.20 | 38.60 | 38.60 | 0.30 | + | + | + | + | + | + | + |
| **Patient 4** | 19 | F | R | 25.22 | 5.32 | 4.57 | 11.7 | 40.0 | 1.70 | 39.70 | 39.90 | 0.52 | + | - | - | + | + | - | + |
| **Patient 4** | 19 | F | L | 25.90 | 5.20 | 4.47 | 12.2 | 39.6 | 1.10 | 39.70 | 39.70 | 0.22 | + | - | - | + | + | - | + |
| **Patient 5** | 56 | F | R | NA | 4.17 | 4.38 | 12.2 | NA | 0.77 | NA | NA | NA | + | + | + | + | + | + | - |
| **Patient 6** | 42 | M | R | 29.60 | 5.17 | 5.22 | 11.7 | 42.1 | 1.60 | 41.80 | 41.70 | 0.70 | - | + | + | + | - | + | - |
| **Patient 6** | 42 | M | L | 29.57 | 5.49 | 5.10 | 12.0 | 41.5 | 2.30 | 41.00 | 40.40 | 0.70 | - | + | + | + | - | + | - |
| **Patient 7** | 8 | M | R | 23.46 | 4.27 | 4.07 | NA | NA | 1.30 | NA | NA | 0.15 | - | + | - | + | + | + | - |
| **Patient 7** | 8 | M | L | 24.02 | 4.42 | 3.85 | 11.8 | NA | 1.00 | NA | NA | 0.10 | - | + | - | + | + | + | - |
| **Patient 8** | 28 | F | R | 25.05 | 4.68 | 4.38 | 13.4 | NA | 1.89 | NA | NA | 0.15 | + | - | - | + | - | + | - |
| **Patient 8** | 28 | F | L | 24.63 | 4.63 | 4.38 | 13.1 | NA | 2.75 | NA | NA | 0.10 | + | - | - | + | - | + | - |

Abbreviations: AL, axial length; ACD, anterior chamber distance; AST, corneal astigmatism; BCVA: best-corrected visual acuity; Cata, cataract; EL, ectopia letis; F, female; Glau, glaucoma; A-Iris, atrophy of the iris; B-Iris, posterior iris bowing; Km, mean corneal keratometry; LogMAR: logarithm of the minimal angle of resolution; L: left; M, male; Meg, megalocornea; NA, not available; R: right; TL, thick lens; WTW: white to white.

**Table S3. Differences in demographic characteristics and various biological parameters between genotype groups.**

|  | **T+T** | **T+M** | **M+M** | **P** |
| --- | --- | --- | --- | --- |
| **Age** | 42.00 [42.00, 42.00] | 15.00 [8.69, 21.31] | - | **0.002** |
| **AL** | 29.59 [29.29, 29.78] | 24.37 [23.82, 24.93] | - | **0.022** |
| **ACD** | 5.33 [3.30, 7.36] | 4.43 [4.13, 4.74] | - | 0.118 |
| **LT** | 5,16 [4.40, 5.92] | 2.95 [1.88, 4.02] | - | **0.034** |
| **WTW** | 11.85 [9.94, 13.76] | 12.23 [11.83, 12.63] | - | 0.599 |
| **Km** | 41.80 [37.99, 45.61] | 40.33 [39.44, 41.23] | - | 0.198 |
| **AST** | 1.95 [-2.50, 6.40] | 1.28 [0.84, 1.72] | - | 0.400 |
| **TCRP (Apex)** | 41.40 [36.32, 46.48] | 39.14 [38.25, 40.03] | - | **0.044** |
| **TCRP(Pupil)** | 41.05 [32.79, 49.31] | 39.23 [38.45, 40.00] | - | **0.044** |

Abbreviations: AL, axial length; ACD, anterior chamber distance; AST, corneal astigmatism; Km, mean corneal keratometry; WTW: white to white; T+T, truncation and truncation variants; T + M, truncation and missense variants; M + M, missense and missense variant.

**Table S4. The *CPAMD8* genotype and phenotype have been published in 3 papers.**

| **PMID** | **ID** | **Nucleotide Changes** | **Protein Changes** | **Group** | **Glaucoma** | **EL** | **Iris** | **Cataract** |
| --- | --- | --- | --- | --- | --- | --- | --- | --- |
| 27839872 | F1 | c.4351T>C | p. Ser1451Pro | 3 | 0 | 0 | 1 | 1 |
|  | F1 | c.4351T>C | p. Ser1451Pro | 3 | 0 | 0 | 1 | 1 |
|  | F2 | c.2352_2353insC | p. Arg785Glnfs*23 | 2 | 0 | 1 | 1 | 1 |
|  | F2 | c.4549-1G>A | p.? | 2 | 0 | 1 | 1 | 1 |
|  | F3-2 | c.700+1G>T | p.? | 3 | 1 | 1 | 1 | 1 |
|  | F3-2 | c.4002+1G>A | p.? | 3 | 1 | 1 | 1 | 1 |
| 32085876 | F1 | c.3403C>T | p. Arg1135ter | 3 | 1 | 1 | 1 | 0 |
|  | F1 | c.3563C>T | p. Pro1188leu | 3 | 1 | 1 | 1 | 0 |
|  | F2 | c.4298C>A | p. Thr1433Asn | 3 | 1 | 1 | 1 | 1 |
|  | F2 | c.1498T>C | p. Cys500Arg | 3 | 1 | 0 | 1 | 1 |
|  | F3 | c.4298C>A | p. Thr1433Asn | 3 | 1 | 0 | 1 | 1 |
|  | F3 | c.1466T>C | p. Leu489Pro | 3 | 1 | 0 | 1 | 1 |
|  | F4 | c.2352dupC | p. Arg785Glnfs*23 | 1 | 1 | 0 | 1 | 0 |
|  | F4 | - | - | 1 | 1 | 0 | 1 | 0 |
|  | F5 | c.2352dupC | p. Arg785Glnfs*23 | 2 | 1 | NA | 1 | 1 |
|  | F5 | c.3231_3232insGGA | p. Ser1078delinsGlyser | 2 | 1 | NA | 1 | 1 |
|  | F6 | c.2449A>T | p. Lys817Ter | 3 | 1 | 0 | 1 | 1 |
|  | F6 | c.2449A>T | p. Lys817Ter | 3 | 1 | 0 | 1 | 1 |
|  | F7 | c.2961dupG | p. Tyr988LeufsTer9 | 3 | 1 | 1 | 1 | 1 |
|  | F7 | c.2961dupG | p. Tyr988LeufsTer9 | 3 | NA | NA | 1 | 1 |
| 32274568 | CG-ASD-160 | c.3686_3687insT | p. Arg1231Profs*64 | 1 | 1 | 0 | 1 | 0 |
|  | CG-ASD-160 | c.1758+1_1758+4del | p. Val1587Serfs*5 | 1 | 1 | 0 | 1 | 0 |
|  | CG-ASD-AN1-0066 | c.2063C>A | p. Ala688Asp | 2 | 1 | 0 | 1 | 1 |
|  | CG-ASD-AN1-0066 | c.2070+4A>C | p. Glu691Valfs*99 | 2 | 1 | 0 | 1 | 1 |
|  | JG-AASD-33 | c.3991G>T | p. Ala1331Ser | 3 | 1 | 0 | 1 | 1 |
|  | JG-AASD-33 | c.1807C>T | p. Pro936Leu | 3 | 1 | 0 | 1 | 1 |
|  | CG-ASD-104 | c.2532del | p. Lys845Argfs*14 | 1 | 1 | 0 | 1 | 0 |
|  | CG-ASD-104 | c.2002C>T | p. Arg668* | 1 | 1 | 0 | 1 | 0 |
|  | PCG-30 | c.1850C>T | p. Ala617Val | 2 | 1 | 0 | 0 | 0 |
|  | PCG-30 | c.4470+10_4470+11insT | p. Trp1494Leufs*5 | 2 | 1 | 0 | 0 | 0 |

EL, ectopia lentis; NA, not available; Group 1, T+T (truncation and truncation variants); Group 2, T + M (truncation and missense variants); Group 3, M + M (missense and missense variant); Ocular complications 1 is positive and 0 is negative.
