# Supplementary material for: Gene jigsaw: Decrypting the CPAMD8 puzzle in Chinese patients with anterior segment dysgenesis
Source: Genes Dis. 2025 Jan 16;12(5):101523. doi: 10.1016/j.gendis.2025.101523 (PMC12163380; doi:10.1016/j.gendis.2025.101523)
Supplement: Multimedia component 2 [file mmc2.docx]

**Methods**

**Patient eligibility and ethics statement**

Patients were recruited in the Eye and ENT Hospital of Fudan University between May 1, 2018 and May 1, 2023. Inclusion criteria were: (1) presence of biallelic *CPAMD8* variant; (2) available relevant clinical information. Exclusion criteria included: (1) coexisting pathogenic variants associated with other ocular diseases; (2) a history of ocular trauma. For the accuracy of the study, only probands in families were included. Ultimately, eight probands (15 eyes) diagnosed with ASD qualified for the study. The left eye of the Patient 5 was excluded due to incomplete data. The study received ethical approval (ChiCTR2000039132) and was conducted following the Declaration of Helsinki's principles, with informed consent obtained from all participants or their legal guardians for minors.

**Ophthalmic examinations**

Comprehensive ophthalmic evaluations were performed, including slit-lamp biomicroscopy, corneal pachymetry, gonioscopy, IOP measurement, spectral-domain optical coherence tomography, and topography analyses (Pentacam HR, Oculus, Wetzlar, Germany). Biometric data were measured with the IOLMaster 700 (Oculus Inc., Wetzlar, Germany). Ciliary body was examined using ultrasound biomicroscopy (MD-300L; Meda Co., Ltd.). All anterior segment comorbidities, including corectopia, pseudopolycoria, corneal opacity, megalocornea, iris hypoplasia, glaucoma, and cataract were confirmed by two experienced ophthalmologists. The “Thick Lens” (TL) condition was defined when lens thickness (LT) exceeded 4.00 mm and the LT/AL ratio (lens thickness to axial length ratio) surpassed 18.0%. Both medical and family histories were documented. The diagnostic criteria of primary glaucoma based on optic nerve damage and visual impairment.

**Genetic and *in silico* analysis**

Peripheral blood sample provided genomic DNA for panel-based Next-Generation Sequencing (NGS) of 289 genes associated with common inherited anterior eye diseases^9^. The methods was previously described by Chen et al^13^.The candidate causal gene variants thereby discovered were confirmed via Sanger sequencing and the primers were designed using the Primer V.3.0 website ((http:// primer3. ut.ee/). Variants were analyzed for biological significance, mapped to protein and genomic structures using the IBS1.0.3 illustrator, and checked against Clinical Genome Resource (Clin Var), Human Genetics Knowledge for the Word (OMIM) and Human Gene Mutation Database (HGMD) for novelty. Pathogenicity predictions for *CPAMD8* variants were assessed using Polyphen2, Mutation Taster and Sorting Intolerant from Tolerant (SIFT), with Silico protein modeling elucidating variant impacts.

**Genotype-phenotype analysis**

Variants were classified as truncation (frameshift, nonsense, splicing) or missense, with truncation variants expected to trigger nonsense-mediated decay. To explore the relationship between gene mutations and ocular complications, comprehensive data on ocular characteristics and comorbidities from both eyes were systematically gathered. Additionally, findings from published non-review literature were incorporated to enrich the analysis.

**Statistical analysis**

Stata MP V. 17.0 (Stata Corp, LLC) and SPSS 20 (IBM Corp., Armonk, NY, USA) were used to conduct all statistical analysis. Continuous variables presented as means ± standard deviation (SD), and categorical variables as counts or proportions. Ocular parameters were compared between groups using Mann-Whitney U-tests or Kruskal-Wallis test. And we applied the Benjamini-Hochberg method to adjust the p-values, controlling the false discovery rate (FDR). Fisher’s exact test or Pearson's chi squared test were employed to assessed the incidences of ocular comorbidities. *P* < 0.05 indicated statistical significance.
